# Supplementary material for: Integrative Taxonomy of Armeria Taxa (Plumbaginaceae) Endemic to Sardinia and Corsica
Source: Plants (Basel). 2023 Jun 5;12(11):2229. doi: 10.3390/plants12112229 (PMC10255103; doi:10.3390/plants12112229)
Supplement: Supplementary file 1 [file plants-12-02229-s001.zip › plants-2405433-supplementary.pdf]

Table S1. Mean and standard deviation of the karyological parameters in *Armeria* taxa endemic to Sardinia and Corsica. Indices names as in the main text; population codes as in Table 1; n = number of studied metaphasic plates; THL = Total Haploid (monoploid) Length, M_{CA} = Mean Centromeric Asymmetry; CV_{CL} = Coefficient of Variation of Chromosome Length; CV_{CI} = Coefficient of Variation of Centromere Index.

Population	n	THL	M_{CA}	CV_{CL}	CV_{CI}	m	sm	st
AR	6	42.21 ± 5.15	15.31 ± 1.43	17.40 ± 3.62	27.90 ± 2.04	46	62	0
BI	7	37.2 ± 4.44	12.1 ± 1.60	18.3 ± 2.03	22.8 ± 2.51	76	50	0
BS	12	38.25 ± 5.76	12.55 ± 1.87	15.52 ± 1.67	25.67 ± 2.54	104	113	0
BU	5	46.89 ± 10.31	15.02 ± 3.61	19.25 ± 3.57	26.65 ± 3.14	44	47	1
CB	9	36.81 ± 4.61	13.34 ± 2.47	17.26 ± 2.65	22.85 ± 1.63	88	76	0
FO	5	42.16 ± 6.08	11.53 ± 1.08	17.35 ± 1.20	25.35 ± 3.37	38	52	0
GO	4	45.25 ± 7.77	14.09 ± 1.69	17.55 ± 1.20	24.35 ± 2.98	38	34	0
MCA	7	44.07 ± 8.61	15.79 ± 1.93	16.48 ± 1.21	22.29 ± 2.50	73	52	0
ML	4	37.66 ± 5.94	14.80 ± 3.18	20.00 ± 3.33	23.49 ± 2.52	40	31	0
MO	8	43.59 ± 6.16	13.59 ± 1.85	18.68 ± 1.09	27.52 ± 2.02	60	83	1
MR	5	38.40 ± 12.85	15.60 ± 3.75	17.38 ± 3.09	22.44 ± 5.56	52	38	0
MS	5	39.22 ± 5.83	13.32 ± 1.11	17.46 ± 1.47	21.72 ± 1.84	58	32	0
RE	12	43.47 ± 4.21	15.08 ± 1.99	16.42 ± 1.34	24.10 ± 1.85	111	106	1
SP	4	41.8 ± 5.05	14.8 ± 1.87	19.7 ± 3.18	24.8 ± 2.88	38	35	1
TH	5	50.9 ± 8.18	13.7 ± 1.10	19.8 ± 2.52	27.7 ± 2.62	36	54	0
Total	91							

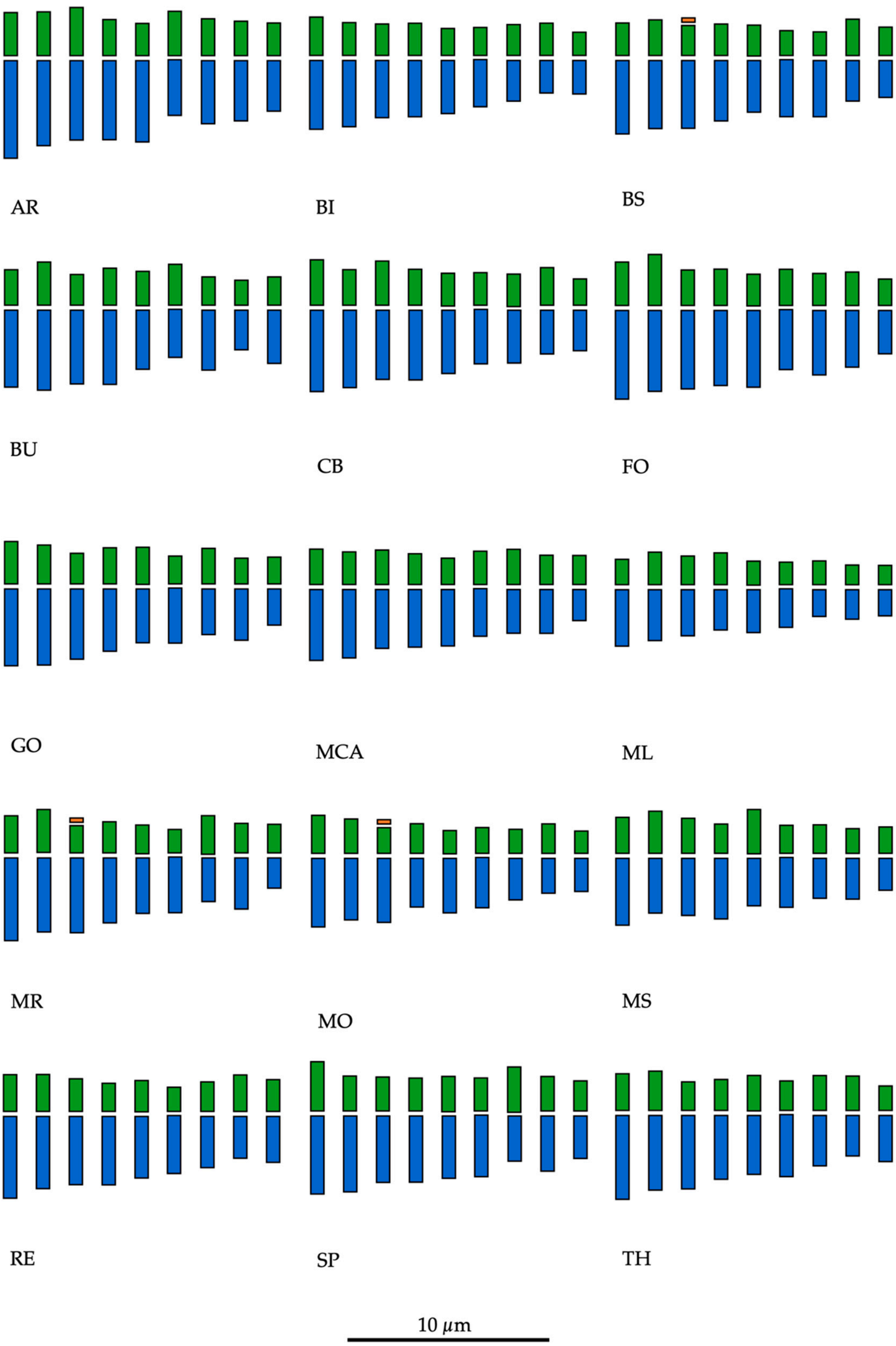

Figure S1. Haploid idiograms of the 15 populations of *Armeria* taxa endemic to Sardinia and Corsica considered in this study. Green = short arm; blue = long arm.

Table S2. Results of IDL tests for all the molecular markers used in the study of *Armeria* taxa endemic to Sardinia and Corsica.

	I (ITS)	H (*trnH-psbA*)	L (*trnL-rpl32*)	Q (*trnQ-rps16*)	S (*trnL-trnF*)	HLSQ (concatenated)
I (ITS)	--	OK(0.1287)	OK(0.9109)	OK(0.4356)	OK(0.1386)	OK(0.7426)

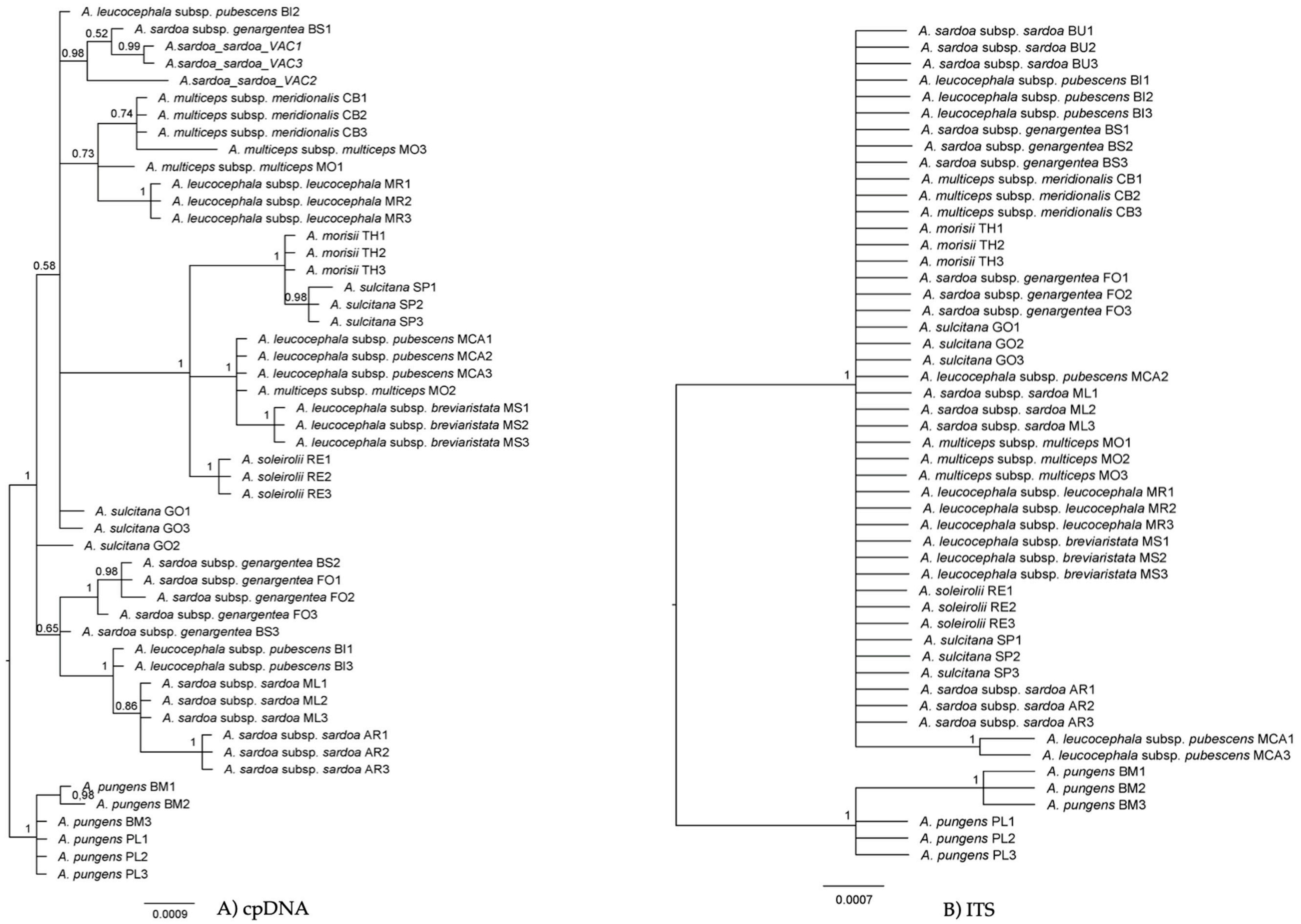

Figure S2. Phylogenetic trees of *Armeria* taxa endemic to Sardinia and Corsica, based on the plastidial markers (*trnH-psbA, trnL-rpl32, trnL-trnF, trnQ-rps16*) (A) and nuclear (ITS) markers (B). Population acronyms as in Figure 1.

Table S3. Mean Decrease Accuracy of Random Forest for each of the 26 seed morphological variables of *Armeria* taxa endemic to Sardinia and Corsica considered in the model.

Variable	Mean Decrease Accuracy
MaxR	76.841325
Concavity	73.343255
Feret	69.508661
MBCRadius	63.924422
CHull	61.394428
Area	59.759716
Shape	59.076689
Perim	58.433220
Pixels	58.325549
PerEquivD	54.647571
CArea	54.630781
Breadth	54.478435
ArEquivD	51.748297
ArBBox	51.102961
EquivEllAr	48.740610
MinR	46.627972
Convexity	37.596910
AspRatio	29.487634
Solidity	27.080916
Circ	13.543970
RFactor	12.157895
Sphericity	7.808802
Roundness	7.179891
Compactness	7.104000
ModRatio	6.009653
Rectang	5.101275

Table S4. List of the 26 seed morphometric features measured in *Armeria* taxa endemic to Sardinia and Corsica.

Features	Description
Perim	Perimeter, calculated from the centres of the boundary pixels
Area	Area inside the polygon defined by the perimeter
Pixels	Number of pixels forming the endocarp image
MinR	Radius of the inscribed circle centred at the middle of the seed
MaxR	Radius of the enclosing circle centred at the middle of the seed
Feret	Largest axis length
Breadth	Largest axis perpendicular to Feret
CHull	Convex hull or convex polygon calculated from pixel centres
CArea	Area of the convex hull polygon
MBCRadius	Radius of the minimal bounding circle
AspRatio	Aspect ratio = Feret/Breadth
Circ	Circularity = $4\pi\times$Area/Perimeter^2
Roundness	Roundness = $4\times$Area/($\pi\times$Feret^2)
ArEquivD	Area equivalent diameter = $\sqrt{((4/\pi)\times \text{Area})}$
PerEquivD	Perimeter equivalent diameter = Area/π
EquivEllAr	Equivalent ellipse area = ($\pi\times$Feret$\cdot$Breadth)/4
Compactness	Compactness = $\sqrt{((4/\pi)\times \text{Area})}$/Feret
Solidity	Solidity = Area/Convex Area
Concavity	Concavity = Convex Area-Area
Convexity	Convexity = Convex hull/Perimeter
Shape	Shape = Perimeter^2/Area
RFactor	RFactor = Convex Hull /(Feret$\times\pi$)
ModRatio	Modification ratio = ($2\times$MinR)/Feret
Sphericity	Sphericity = MinR/MaxR
ArBBox	Area of the bounding box along the feret diameter = Feret$\cdot$Breadth
Rectang	Rectangularity = Area/ArBBox

Table S5. Descriptive statistics of the five most important seed morphometric features in *Armeria* taxa endemic to Sardinia and Corsica, found by Random Forest.

Population	MaxR	Concavity	Feret	MBCRadius	CHull
AR	55.7 ± 4.3	131.3 ± 123.7	107.6 ± 7.6	53.8 ± 3.8	246.4 ± 15.4
BS	50.7 ± 4.7	164.5 ± 78.6	96.9 ± 10.0	48.5 ± 4.8	226.8 ± 18.2
BU	52.5 ± 11.1	155.8 ± 195.3	99.2 ± 21.4	49.6 ± 10.7	225.8 ± 41.3
CB	58.1 ± 4.1	110.4 ± 59.1	111.7 ± 7.6	55.9 ± 3.8	253.6 ± 14.0
FO	16.7 ± 0.8	14.8 ± 3.4	32.4 ± 1.5	16.2 ± 0.7	74.3 ± 2.6
GO	56.8 ± 4.7	129.6 ± 100.3	109.1 ± 8.1	54.5 ± 4.0	250.1 ± 17.2
MCA	61.2 ± 7.8	172.3 ± 595.4	116.4 ± 11.7	58.2 ± 5.9	263.0 ± 26.7
ML	51.8 ± 6.1	133.6 ± 244.4	99.3 ± 11.5	49.6 ± 5.8	227.6 ± 26.9
MO	59.2 ± 6.4	190.0 ± 238.2	113.9 ± 12.1	56.9 ± 6.0	258.9 ± 25.3
MR	60.3 ± 5.1	115.3 ± 82.1	114.6 ± 9.6	57.3 ± 4.8	260.9 ± 20.4
MS	55.1 ± 3.9	96.6 ± 45.3	106.0 ± 6.9	53.0 ± 3.5	241.2 ± 14.5
RE	50.8 ± 4.3	241.8 ± 222.2	97.3 ± 8.1	48.7 ± 4.0	224.9 ± 18.8
SP	16.5 ± 1.5	15.6 ± 4.2	31.7 ± 3.0	15.9 ± 1.5	73.2 ± 5.7
TH	59.3 ± 3.5	195.8 ± 192.6	116.5 ± 6.8	58.3 ± 3.4	270.7 ± 16.8

Table S6. Confusion matrix from the tuned Random Forest model on the current taxonomic hypothesis for the seed morphometric data in *Armeria* taxa endemic to Sardinia and Corsica.

	A. leucocephala subsp. *breviaristata*	*A. leucocephala* subsp. *leucocephala*	*A. leucocephala* subsp. *pubescens*	*A. morisii*	*A. multiceps* subsp. *meridionalis*	*A. multiceps* subsp. *multiceps*	*A. sardoa* subsp. *genargentea*	*A. sardoa* subsp. *sardoa*	*A. soleirolii*	*A. sulcitana*	Class Error
A. leucocephala subsp. *breviaristata*	**32**	9	10	0	9	2	1	25	1	11	0.68
A. leucocephala subsp *leucocephala*	8	**34**	26	2	8	5	0	13	1	3	0.66
A. leucocephala subsp. *pubescens*	15	25	**39**	2	19	14	0	20	1	8	0.72
A. morisii	0	3	2	**65**	3	4	0	7	1	8	0.30
A. multiceps subsp. *meridionalis*	13	2	24	5	**23**	7	0	16	0	10	0.77
A. multiceps subsp. *multiceps*	8	5	22	7	13	**20**	3	10	8	6	0.80
A. sardoa subsp. *genargentea*	0	0	0	1	0	0	**51**	11	6	28	0.47
A. sardoa subsp. *sardoa*	22	6	9	9	15	3	1	**296**	31	14	0.27
A. soleirolii	1	0	2	0	0	1	0	40	**59**	1	0.43
A. sulcitana	12	3	6	11	9	6	27	19	3	**104**	0.48

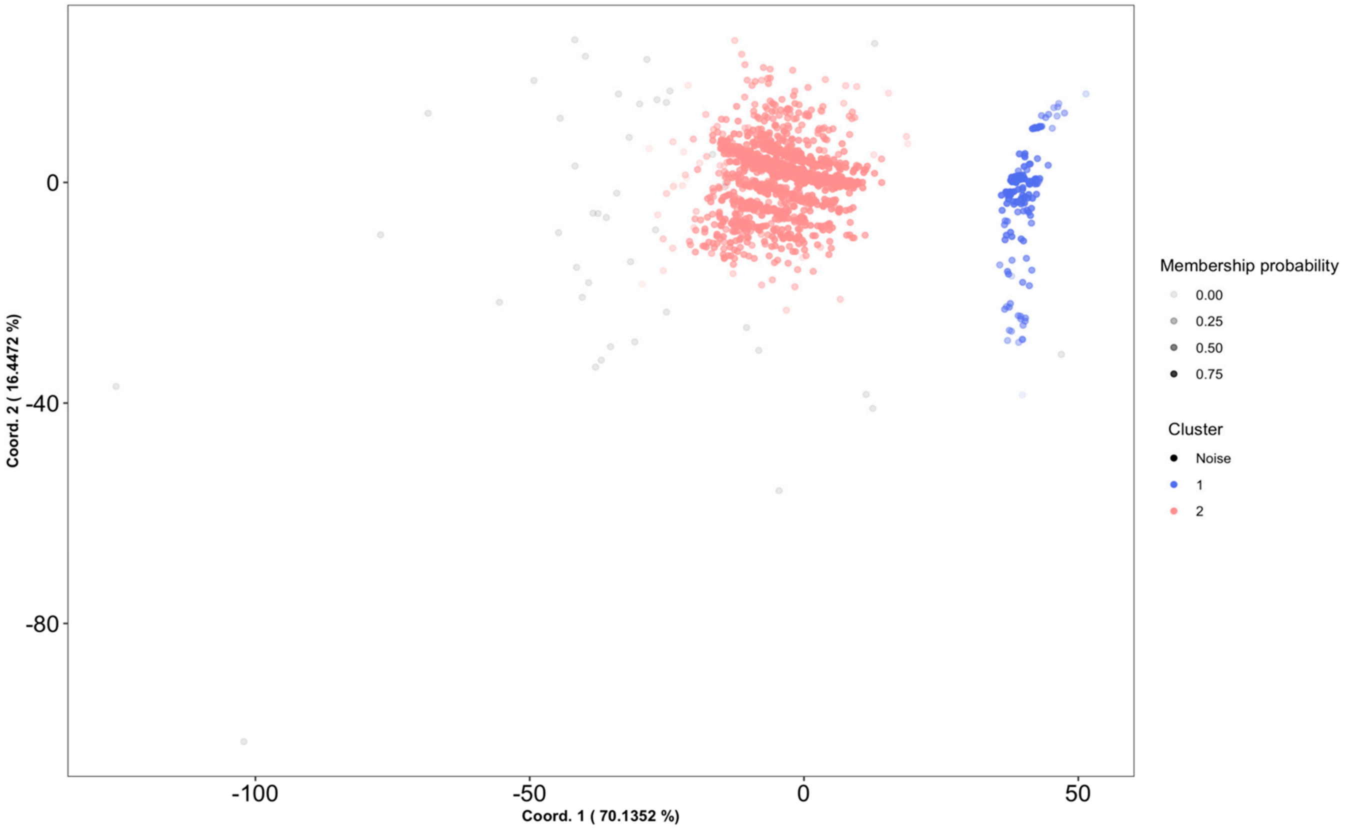

Figure S3. PCoA based on the Manhattan distance of the 26 seed morphometric features in *Armeria* taxa endemic to Sardinia and Corsica, colored based on the results of HDBSCAN* clustering algorithm. The first two axes of PCoA retained more than 86% of the variation. Likelihood of belonging to one of the two cluster, estimated from the PDF obtained by HBSCAN*, is also represented as transparency of the points. The 42 seeds considered as potential outliers are also represented.

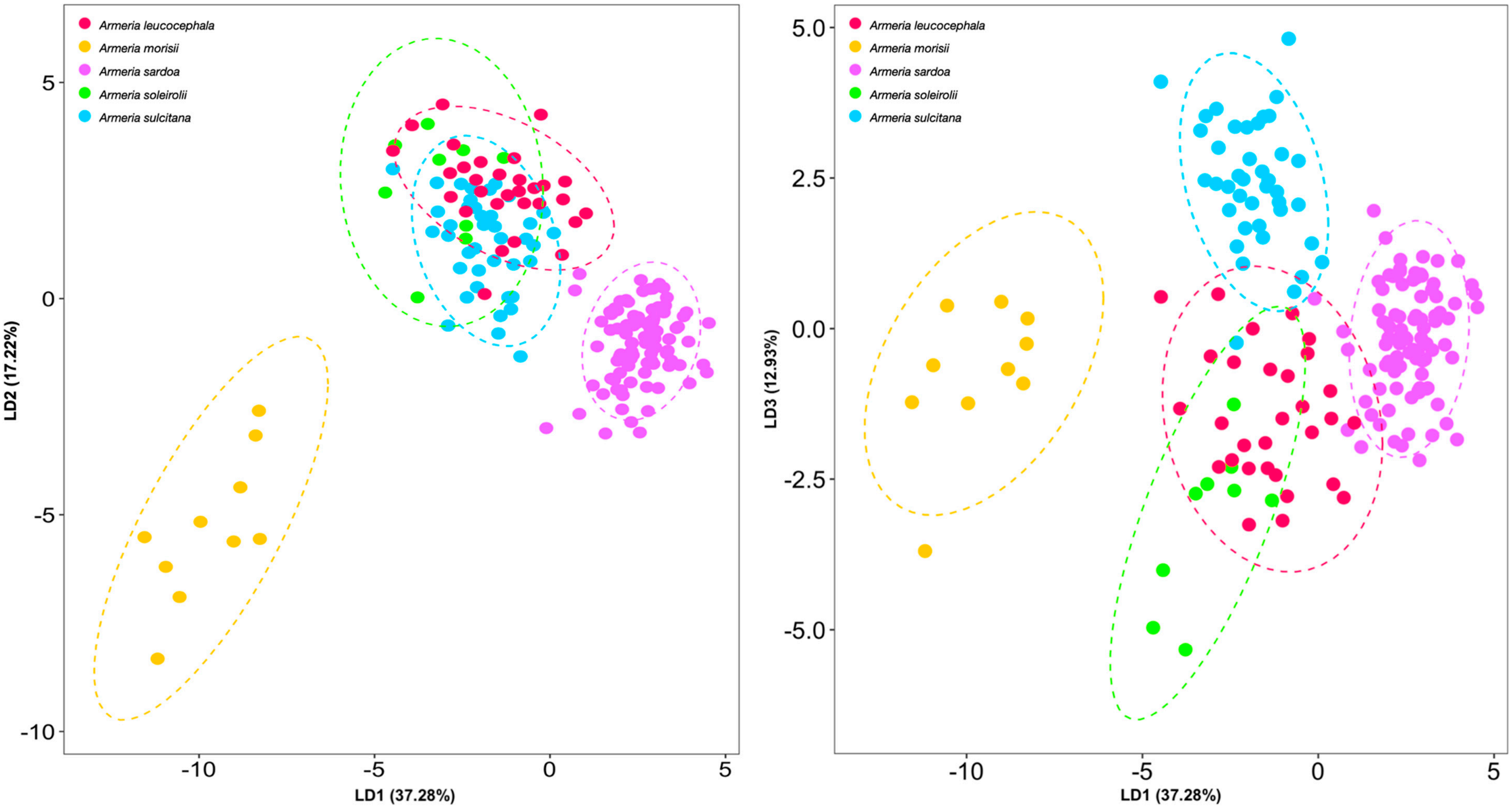

Figure S4. Canonical Variate Analysis of the morphometric dataset of *Armeria* taxa endemic to Sardinia and Corsica, composed by numeric variables grouped based on the new taxonomic circumscription. 95% Confidence Ellipses are drawn assuming a *t*-distribution of points.

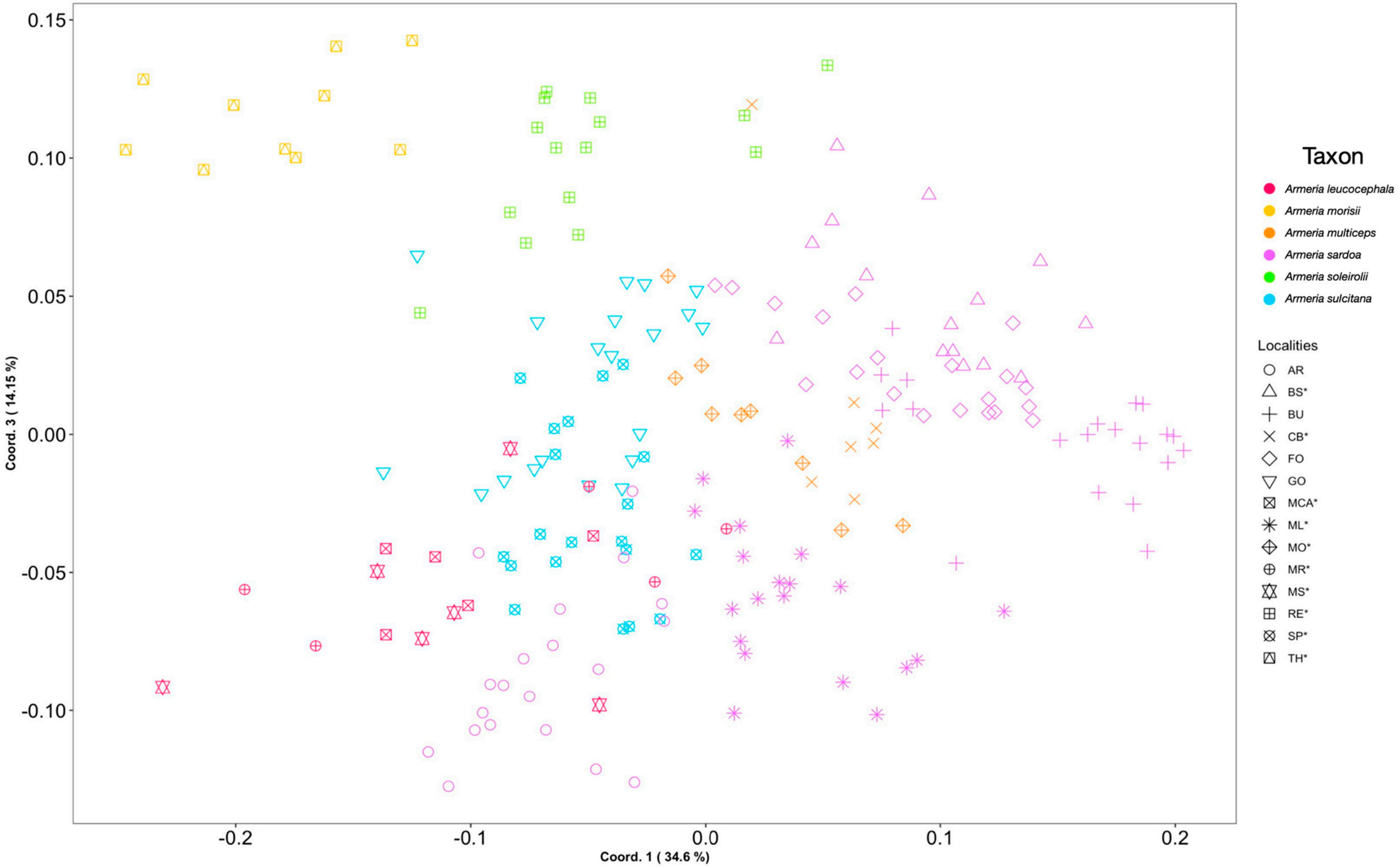

Figure S5. PCoA illustrating the morphometric variation of the first and third axes in *Armeria* taxa endemic to Sardinia and Corsica, based on the Gower distance of the 49 characters measured. Symbols indicates the acronym of the population localities (Table 1), whereas colours indicate the taxa at species level. Asterisk = type localities.

Table S7. Confusion matrix for the species of *Armeria* taxa endemic to Sardinia and Corsica, based on the current taxonomic hypothesis.

	A. leucocephala subsp. *breviaristata*	*A. leucocephala* subsp. *leucocephala*	*A. leucocephala* subsp. *pubescens*	*A. morisii*	*A. multiceps* subsp. *meridionalis*	*A. multiceps* subsp. *multiceps*	*A. sardoa* subsp. *sardoa*	*A. sardoa* subsp. *genargentea*	*A. soleirolii*	*A. sulcitana*
A. leucocephala subsp. *breviaristata*	**2**	2	1	0	0	0	0	0	0	0
A. leucocephala subsp. *leucocephala*	0	**1**	0	0	0	0	0	0	0	0
A. leucocephala subsp. *pubescens*	1	1	**4**	0	0	0	0	0	0	0
A. morisii	0	0	0	**10**	0	0	0	0	0	0
A. multiceps subsp. *meridionalis*	0	0	0	0	**6**	1	0	0	0	0
A. multiceps subsp. *multiceps*	0	1	0	0	0	**7**	0	0	0	0
A. sardoa subsp. *sardoa*	0	0	0	0	0	0	**35**	1	0	0
A. sardoa subsp. *genargentea*	0	0	0	0	0	1	0	**55**	0	0
A. soleirolii	0	0	0	0	0	0	0	0	**9**	0
A. sulcitana	2	0	0	0	0	0	0	2	0	**40**

Table S8. Confusion matrix of the morphometric data in *Armeria* taxa endemic to Sardinia and Corsica, according to the tuned kNN model under the alternative taxonomic hypothesis 1 (6 species, no subspecies).

	A. leucocephala	*A. morisii*	*A. multiceps*	*A. sardoa*	*A. soleirolii*	*A. sulcitana*
A. leucocephala	**12**	0	0	0	0	0
A. morisii	0	**10**	0	0	0	0
A. multiceps	2	0	**15**	0	0	0
A. sardoa	0	0	0	**91**	0	0
A. soleirolii	0	0	0	0	**9**	0
A. sulcitana	1	0	0	2	0	**40**

Table S9. Confusion matrix of the morphometric data in *Armeria* taxa endemic to Sardinia and Corsica, according to the tuned kNN model under the alternative taxonomic hypothesis 2 (five species, no subspecies).

	A. leucocephala	*A. morisii*	*A. sardoa*	*A. soleirolii*	*A. sulcitana*
A. leucocephala	**28**	0	0	0	0
A. morisii	0	**10**	0	0	0
A. sardoa	0	0	**92**	0	0
A. soleirolii	0	0	0	**9**	0
A. sulcitana	2	0	1	0	**40**

Table S10. Descriptive statistics for the continuous characters according to the new taxonomic circumscription of *Armeria* taxa endemic to Sardinia and Corsica. Some individuals have been removed due to the presence of missing values.

Species	n	HEIGTH	SCAP_NUM	SCA_LENG	SCA_DIAM	WIDTH_IAL_WIN	ANG_WIN_TIP	LENG_WIN_LEAF	WIDTH_WIN_LEAF
A. leucocephala	30	195.74 ± 93.75	4.03 ± 3.10	155.37 ± 86.45	1.05 ± 0.35	0.04 ± 0.02	20.75 ± 2.38	36.71 ± 23.78	1.11 ± 0.40
A. morisii	10	275.08 ± 29.39	3.60 ± 1.43	230.01 ± 46.84	1.34 ± 0.21	0.09 ± 0.04	52.64 ± 4.13	39.85 ± 19.44	5.08 ± 1.31
A. sardoa	93	200.91 ± 90.02	7.83 ± 5.03	163.54 ± 76.46	0.92 ± 0.25	0.03 ± 0.02	27.69 ± 9.22	28.19 ± 17.48	1.75 ± 0.63
A. soleirolii	9	195.00 ± 44.06	2.67 ± 1.50	168.67 ± 41.18	0.86 ± 0.17	0.08 ± 0.13	23.20 ± 6.04	36.39 ± 19.04	1.69 ± 0.37
A. sulcitana	40	300.62 ± 41.19	7.58 ± 4.70	257.61 ± 41.80	1.22 ± 0.22	0.02 ± 0.02	24.61 ± 4.40	35.12 ± 9.97	1.71 ± 0.54

Species	n	WIDTH_IAL_SUM	ANG_SUM_TIP	LENG_SUM_LEAF	WIDTH_SUM_LEAF	N_SUM_VEINS	SHEATH_LENG	DIAM_CAP	LENG_OUT_INV_BRACT
A. leucocephala	30	0.03 ± 0.02	21.63 ± 3.28	42.34 ± 26.24	1.25 ± 0.38	2.50 ± 0.94	8.94 ± 3.33	14.01 ± 3.77	4.89 ± 1.11
A. morisii	10	0.07 ± 0.03	44.02 ± 6.02	46.02 ± 14.47	4.98 ± 1.31	3.60 ± 0.97	12.98 ± 2.79	18.42 ± 2.66	7.07 ± 1.06
A. sardoa	93	0.03 ± 0.03	19.45 ± 4.42	38.68 ± 23.30	1.10 ± 0.37	2.66 ± 0.96	8.07 ± 2.27	11.43 ± 2.70	4.95 ± 0.89
A. soleirolii	9	0.03 ± 0.01	31.53 ± 11.47	46.03 ± 15.02	1.71 ± 0.58	3.00 ± 0.00	9.67 ± 1.71	15.66 ± 1.77	3.72 ± 0.75
A. sulcitana	40	0.02 ± 0.02	19.26 ± 1.56	51.99 ± 16.15	1.34 ± 0.49	3.20 ± 0.76	9.95 ± 3.17	13.21 ± 1.76	4.83 ± 0.76

Table S10. *Cont.*

Species	n	WIDTH_OUT_INV_BRACT	LENG_INTER_INV_BRACT	WIDTH_INTER_INV_BRACT	LENG_INNER_INV_BRACT	WIDTH_INNER_INV_BRACT	N_INV_BRACT	LENG_OUTER_SPI_BRACT	WIDTH_OUTER_SPI_BRACT	LENG_OUTER_SPI_BRACLE	WIDTH_OUTER_SPI_BRATLE
A. leucocephala	30	2.75 ± 0.76	5.71 ± 1.45	2.77 ± 0.83	6.54 ± 1.23	2.82 ± 0.48	7.67 ± 1.60	7.17 ± 1.07	4.45 ± 0.71	5.00 ± 0.85	3.29 ± 0.84
A. morisii	10	3.84 ± 0.49	10.12 ± 1.29	4.58 ± 0.70	9.70 ± 0.70	4.03 ± 0.69	9.30 ± 1.49	9.50 ± 0.98	5.71 ± 0.55	6.13 ± 0.52	4.29 ± 0.42
A. sardoa	93	2.59 ± 0.81	4.78 ± 0.99	2.24 ± 0.65	3.73 ± 0.68	1.78 ± 0.54	8.65 ± 1.50	5.91 ± 0.93	3.24 ± 0.81	4.09 ± 0.78	2.53 ± 0.61
A. soleirolii	9	2.09 ± 0.35	4.53 ± 0.65	3.49 ± 0.61	6.43 ± 1.07	3.76 ± 0.52	9.44 ± 1.59	6.85 ± 0.50	4.81 ± 0.78	4.91 ± 0.67	3.28 ± 0.37
A. sulcitana	40	2.52 ± 0.47	6.37 ± 1.31	3.00 ± 0.54	6.75 ± 1.00	3.08 ± 0.73	9.32 ± 2.08	6.96 ± 0.65	4.45 ± 0.72	4.53 ± 0.48	2.91 ± 0.60

Species	n	LENG_INNER_SPI_BRACT	WIDTH_INNER_SPI_BRACT	LENG_INNER_SPI_BRACLE	WIDTH_INNER_SPI_BRACLE	LENG_CAL_PED	LENG_CAL_TUBE	WIDTH_CAL_TUBE	LIMB_LENG	AWN_LENG
A. leucocephala	30	6.63 ± 1.24	4.12 ± 1.13	4.47 ± 0.84	2.74 ± 0.52	1.35 ± 0.30	2.58 ± 0.45	1.32 ± 0.32	2.38 ± 0.40	0.63 ± 0.27
A. morisii	10	8.97 ± 0.96	4.86 ± 0.36	6.12 ± 0.94	3.74 ± 0.42	1.36 ± 0.22	2.74 ± 0.29	1.06 ± 0.10	2.45 ± 0.23	1.31 ± 0.18
A. sardoa	93	5.50 ± 0.88	3.11 ± 0.63	3.84 ± 0.82	2.41 ± 0.47	1.15 ± 0.35	2.31 ± 0.51	1.26 ± 0.42	2.01 ± 0.50	0.44 ± 0.16
A. soleirolii	9	7.41 ± 0.75	4.84 ± 0.73	5.45 ± 0.94	3.37 ± 0.35	1.33 ± 0.30	2.59 ± 0.21	1.19 ± 0.39	1.88 ± 0.59	0.62 ± 0.08
A. sulcitana	40	6.30 ± 0.69	3.75 ± 0.53	4.13 ± 0.57	2.61 ± 0.55	1.39 ± 0.42	2.25 ± 0.28	1.23 ± 0.25	1.91 ± 0.36	0.77 ± 0.17

Table S11. Contingency tables of most significant categorical characters used in the identification key to *Armeria* taxa endemic to Sardinia and Corsica. Significance according to Fisher's exact test.

PAP_CELL	*A. leucocephala*	*A. morisii*	*A. sardoa*	*A. soleirolii*	*A. sulcitana*
Yes	0	0	0	15	0
No	32	10	95	0	40
Significance	a	a	a	b	a

DIMORF	*A. leucocephala*	*A. morisii*	*A. sardoa*	*A. soleirolii*	*A. sulcitana*
Homomorphic	31	10	18	9	10
Dimorphic	1	0	77	6	30
Significance	a	a	b	a	b

MAR_SUM_LEAF	*A. leucocephala*	*A. morisii*	*A. sardoa*	*A. soleirolii*	*A. sulcitana*
Smooth	18	10	44	15	40
Dentate	14	0	51	0	0
Significance	a	b	a	b	b

CALYX_HAIRINESS	*A. leucocephala*	*A. morisii*	*A. sardoa*	*A. soleirolii*	*A. sulcitana*
Holotrichous	1	10	35	0	0
Pleurotrichous	30	0	60	9	40
Significance	a	b	c	a	a

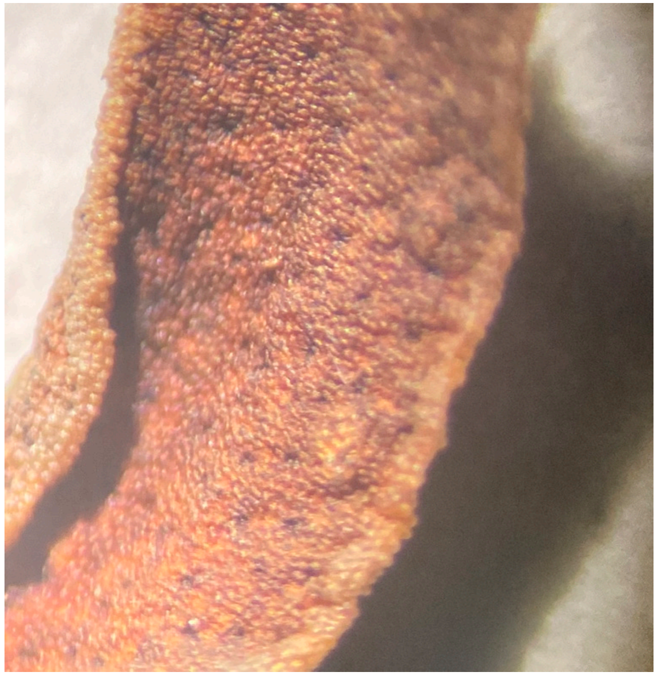

Figure S6. Leaf surface of *Armeria soleirolii* and its papillate epidermal cells.
